# Monitoring Insecticide Susceptibility in *Aedes Aegypti* Populations from the Two Biggest Cities, Ouagadougou and Bobo-Dioulasso, in Burkina Faso: Implication of Metabolic Resistance

**DOI:** 10.3390/tropicalmed5020084

**Published:** 2020-05-27

**Authors:** Moussa Namountougou, Dieudonné Diloma Soma, Mahamoudou Balboné, Didier Alexandre Kaboré, Mahamadi Kientega, Aristide Hien, Ahmed Coulibaly, Parfait Eric Ouattara, Benson Georges Meda, Samuel Drabo, Lassane Koala, Charles Nignan, Thérèse Kagoné, Abdoulaye Diabaté, Florence Fournet, Olivier Gnankiné, Roch Kounbobr Dabiré

**Affiliations:** 1 Institut de Recherche en Sciences de la Santé (IRSS), Bobo-Dioulasso BP 545, Burkina Faso; ddsoma@irss-dro.bf (D.D.S.); dakabore@irss-dro.bf (D.A.K.); mkientega@irss-dro.bf (M.K.); ahien@irss-dro.bf (A.H.); peric.ouattara@centre-muraz.bf (P.E.O.); bgmeda@irss-dro.bf (B.G.M.); lkoala@irss-dro.bf (L.K.); cnignan@irss-dro.bf (C.N.); adiabate@ic.ac.uk (A.D.); 2 Département des Sciences Biomédicales, Centre Muraz, Bobo-Dioulasso BP 390, Burkina Faso; therese.kagone@centre-muraz.bf; 3 Institut Supérieur des Sciences de la Santé, Université Nazi Boni, Bobo-Dioulasso BP 1091, Burkina Faso; 4 Département de Biologie et de Physiologie Animales, Université Joseph Ki-Zerbo, Ouagadougou BP 7021, Burkina Faso; mahamoudou.balbone@univ-ouaga.bf (M.B.); samuel.drabo@univ-ouaga.bf (S.D.); olivier.gnankine@univ-ouaga.bf (O.G.); 5 Unité de Formation et de Recherche en Sciences et Techniques, Université Norbert Zongo, Koudougou BP 376, Burkina Faso; ahmed.coulibaly@univ-ouaga.bf; 6 Maladies Infectieuses et Vecteurs: Écologie et Contrôle (MIVEGEC), Univ Montpellier, CNRS, IRD, 34394 Montpellier, France; florence.fournet@ird.fr

**Keywords:** *Aedes aegypti*, dengue, insecticide susceptibility, metabolic resistance, Burkina Faso

## Abstract

In West Africa, *Aedes aegypti* remains the major vector of dengue virus. Since 2013, dengue fever has been reemerging in Burkina Faso with annual outbreaks, thus becoming a major public health problem. Its control relies on vector control, which is unfortunately facing the problem of insecticide resistance. At the time of this study, although data on phenotypic resistance were available, information related to the metabolic resistance in *Aedes* populations from Burkina Faso remained very scarce. Here, we assessed the phenotypic and the metabolic resistance of *Ae. aegypti* populations sampled from the two main urban areas (Ouagadougou and Bobo-Dioulasso) of Burkina Faso. Insecticide susceptibility bioassays to chlorpyriphos-methyl 0.4%, bendiocarb 0.1% and deltamethrin 0.05% were performed on natural populations of *Ae. aegypti* using the WHO protocol. The activity of enzymes involved in the rapid detoxification of insecticides, especially non-specific esterases, oxidases (cytochrome P450) and glutathione-S-transferases, was measured on individual mosquitos. The mortality rates for deltamethrin 0.05% were low and ranged from 20.72% to 89.62% in the Bobo-Dioulasso and Ouagadougou sites, respectively. When bendiocarb 0.1% was tested, the mortality rates ranged from 7.73% to 71.23%. Interestingly, in the two urban areas, mosquitoes were found to be fully susceptible to chlorpyriphos-methyl 0.4%. Elevated activity of non-specific esterases and glutathione-S-transferases was reported, suggesting multiple resistance mechanisms involved in *Ae. aegypti* populations from Bobo-Dioulasso and Ouagadougou (including cytochrome P450). This update to the insecticide resistance status within *Ae. aegypti* populations in the two biggest cities is important to better plan dengue vectors control in the country and provides valuable information for improving vector control strategies in Burkina Faso, West Africa.

## 1. Background

Dengue is now the most important emerging mosquito viral disease and constitutes a major public health threat in disease-endemic regions [1]. Up to now, four serotypes (DENV1, DENV2, DENV3, DENV4) have been found to be responsible for dengue disease. Dengue virus (*Flaviviridae, Flavivirus*) is transmitted during infected bites of *Aedes* female mosquitoes [1]. In Africa, *Aedes aegypti* remains one of the main dengue virus vectors [2]. In Burkina Faso, *Ae. aegypti* is assumed to be the main dengue vector in urban areas [3,4] and is characterized by a diurnal and crepuscular activity [5]. It is also most frequently identified at larval stages from breeding sites such as water containers and used tires [4]. 

In Burkina Faso, a dengue epidemic was first reported in 1925 [6] and then in 1983 [7]. Outbreaks were reported in Ouagadougou, the biggest city of Burkina Faso in 2013, 2015, and 2016 [1]. DENV2 was found in a number of cases in the 1980s, while DENV1 was found among travelers returning from Burkina Faso in the 2000s [8]. The presence of DENV3 was also observed in an European patient who had travelled in Burkina Faso [9]. In Ouagadougou, DENV2, DENV3, DENV4 were reported during a cross sectional survey done from December 2013 to January 2014 [4]. In 2016, more than 1061 cases of dengue and 15 deaths were recorded in Ouagadougou [1]. In this city, *Ae. aegypti* populations were found in artificial water containers such as flower pots, buckets, gutters, and tires, which are associated with human activities. 

Sound knowledge on vector species bio-ecology is essential for the implementation of vector control strategies as no vaccine is available. Among the methods currently used, environmental management, mechanic elimination of the breeding habitats, chemical use and to, a lesser extent, biological agents, might be efficient against *Aedes* populations and consequently in reducing the incidence of the disease. 

Recent reports of insecticide resistance in dengue vector populations in Burkina Faso revealed that *Ae. aegypti* was resistant to pyrethroid (deltamethrin 0.05% and permethrin 0.75%) and bendiocarb 0.1%, whereas it was fully susceptible to malathion 5% [10]. However, there are no available data upon the implication of detoxification enzymes in insecticide resistance within *Ae. aegypti* populations in Burkina Faso. This study aimed to explore this gap, evaluating the resistance level of *Ae. aegypti* populations to the three classes of insecticide used in public health in Burkina Faso. 

## 2. Methods

### 2.1. Study Area

**Figure 1:**
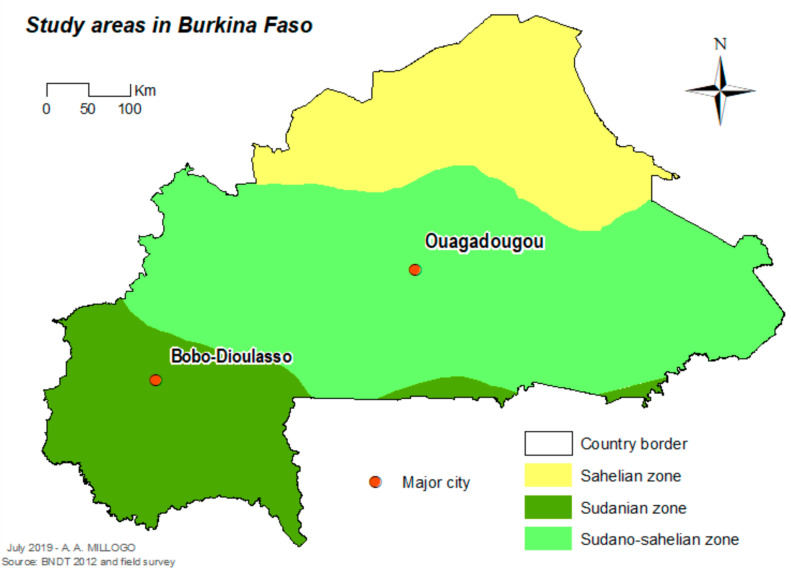
Localization of the study cities in Burkina Faso.

The study was carried out in Ouagadougou (12°21′56″ N, 1°32′01″ W), the capital city in the central part of the country, and Bobo-Dioulasso (11°10′59″ N, 4°16′59″ W) the second biggest city of the country in the western part of the country (Figure 1). In 2016, the population was estimated as 2,293,635 inhabitants in Ouagadougou and 780,846 inhabitants in Bobo-Dioulasso [11] Ouagadougou is located in the Sudan-Sahelian area with a short rainy season extending from June to September and an average yearly rainfall of between 600 and 900 mm. Bobo-Dioulasso is located in the Sudan savannah zone. The rainy season extends from May to November with an average yearly rainfall above 900 mm. 

### 2.2. Mosquito Collection and Rearing

We collected *Ae. aegypti* mosquito larvae in the years 2013, 2014 and 2016 in a random sample of abandoned tires (twenty per site per year), found close to human dwellings. In each sample of abandoned tires, 80–100 larvae were collected and then brought back in the insectary of IRSS/DRO in Bobo-Dioulasso, where they were reared under standard controlled conditions (25 ± 2 °C, 80 ± 10% RH and 12:12 L-D) until adulthood. Mosquitoes were identified using morphological keys [12,13]. Adult mosquitoes issued from field collections (F0), fed with 5% sugar solution, were subsequently used for the insecticide susceptibility tests. The susceptible strain of *Ae. aegypti,* received from Montpellier and maintained in the insectary of IRSS, was used as control for insecticide bioassays. 

### 2.3. Insecticide Susceptibility Tests

Insecticide susceptibility tests were performed on 3–5 days old females of *Ae. aegypti* using WHO diagnostic doses on mosquitoes collected in 2013, 2014 and 2016 [14]. The bioassays were done with papers impregnated with deltamethrin 0.05%, bendiocarb 0.1% and chlorpyrifos-methyl 0.4%. The wild F0 and the susceptible strain of *Ae. aegypti* populations were exposed to the same papers. For each insecticide paper, four replicates of 20–25 *Ae. aegypti* were exposed for 1 h. For each assay, control mosquitoes were also exposed to papers only impregnated with silicon oil following the same procedure. Mortality rates were recorded 24 h after insecticide exposure. 

### 2.4. Enzyme Activities

Biochemical assays were performed to check the activity of detoxifying enzymes, namely non-specific esterases (NSE), mixed-function oxidases (MFO) and glutathione-S-transferases (GST) involved in insecticide resistance within *Ae*. *aegypti* mosquito populations. The assays were conducted on 3–5 days old *Ae. aegypti* adults issued from field collections (F0) (not previously exposed to insecticides and stored at −80 °C) using standard protocol [15]. Experimental procedures are detailed in Namountougou *et al.* [16,17]. 

### 2.5. Statistical Analyses

Mortality rates recorded during bioassays were analyzed according to the WHO criteria [18]. The populations of *Ae. aegypti* were classified as “resistant” if less than 90% mortality was observed, as “suspected resistant” if mortality rates were between 90% and 98% and “susceptible” for more than a 98% mortality rate. Biochemical data were analyzed and compared between the two groups (wild and susceptible strains) using non-parametric Mann–Whitney test run with Graph Pad Prism 5 software. 

## 3. Results

### 3.1. Insecticide Susceptibility Test

**Figure 2:**
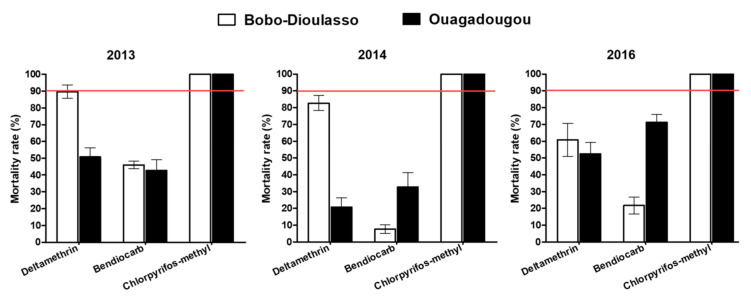
Mortality rates of the *Aedes aegypti* populations in Ouagadougou and Bobo-Dioulasso during the three years of survey. Red bars indicate the threshold of susceptibility.

The mortality rate in unexposed controls of wild and susceptible adult mosquito strain was less than 5% and no correction of the mortality rate was then required (Figure 2). Tests performed with deltamethrin in 2013 and 2014 on *Ae. aegypti* populations collected in Bobo-Dioulasso showed mortality rates (89.62% and 82.72%, respectively) lower than 90%, suggesting a resistance. Mortalities of *Ae. aegypti* collected in Ouagadougou were 50.7% and 20.7% for 2013 and 2014, respectively, when exposed to deltamethrin, showing a high resistance level. Two years later, in 2016, *Ae. aegypti* populations from Bobo-Dioulasso and Ouagadougou exhibited resistance to deltamethrin, with 60.72% and 52.54% mortality rates, respectively. 

During the three-year survey, *Ae. aegypti* populations from Ouagadougou and Bobo-Dioulasso were found to be fully resistant to bendiocarb 0.1% and the mortality rates ranged from 7.73% to 71.23%. Inversely, all *Ae. aegypti* populations tested in 2013, 2014 and 2016 against chlorpyriphos-methyl 0.4% were fully susceptible, with 100% of mortality rates in both cities. 

### 3.2. Non-Specific Esterases (NSE) and Para Nitro Phenyl Acetate (PNPA)

**Figure 3:**
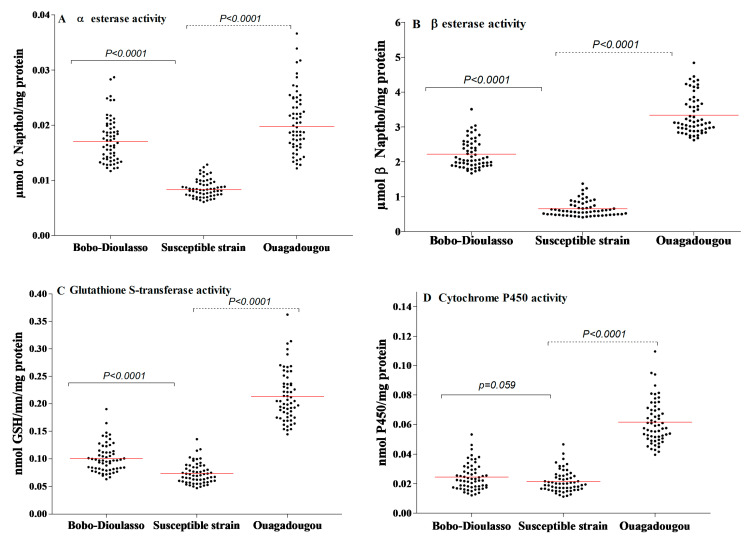
Detoxifying enzyme activities in *Aedes aegypti* mosquitoes collected in Bobo-Dioulasso and Ouagadougou in 2016. (**A**) Non-specific αEsterase activity, (**B**) PPNA activity, (**C**) glutathione-S-transferase (GST) activity, (**D**) P450 activity. P-value denotes significant difference in activity level when compared to the *Ae. aegypti* (from Montpellier) reference strain according to the Mann–Whitney U test. Red bars indicate the median value in each strain.

The results of NSE and PNPA activities are shown in Figure 3A,B. The NSE activity within the susceptible strain of *Ae. aegypti* ranged from 0.006 to 0.012 mmol NSE/mg protein (the median activity was 0.008). Concerning the PNPA, its activity was also high and varied from 0.410 to 1.378 mmol PNPA/mg protein (the median activity was 0.585). For both cities, the results show higher levels of esterase activity compared to the susceptible strain (Mann–Whitney test, *p* < 0.0001). 

### 3.3. Gluthathione-S-Transferases (GST)

The levels of GST activity within the laboratory and the field populations are shown in Figure 3C. The susceptible strain showed a GST activity ranging from 0.047 to 0.135 mmol GSH/min/mg protein (the median activity was 0.069). The activity level of GST within the wild populations was higher than that recorded in the susceptible strain (Mann–Whitney test, *p* < 0.0001 in both cities). 

### 3.4. Oxidases (Cytochrome P450)

The activity level of oxidase (P450) in the susceptible and wild strains is shown in Figure 3D. Oxidase activity in the susceptible strain ranged from 0.0112 to 0.0465 nmol P450/mg protein (the median activity was 0.020). A significantly higher level of P450 activity was detected in wild populations from Ouagadougou (Mann–Whitney test, p < 0.0001). However, there was no statistically difference between field and susceptible strains in Bobo-Dioulasso (Mann–Whitney test, *p* = 0.059). 

## 4. Discussion

*Aedes aegypti* is identified as the main dengue vector in Burkina Faso [10]. The knowledge about its susceptibility to insecticides is essential for the implementation of the suitable control of *Ae*. *aegypti* populations, especially during dengue outbreaks requiring a rapid riposte. In this study, the surveillance activities were limited to the two largest cities in the country, where dengue outbreaks have been regularly reported since 2013 [19]. *Aedes aegypti* populations collected in Bobo-Dioulasso and Ouagadougou were found to be fully resistant to deltamethrin and bendiocarb during the two first years (2013 and 2014) of monitoring. Bioassays carried out in 2013 indicated moderate resistance level with deltamethrin and reduced mortality, averaging 80% in 2014 in Bobo-Dioulasso. Then, *Ae. aegypti* was found to be fully resistant to deltamethrin in 2016, with mortality rates below 61%. This resistance status was already notified in 2015 by Ouattara et al. [10] along a transect from Banfora to Ouagadougou. 

The resistance in the *Ae. aegypti* populations might be selected by the high use of insecticides in households such as aerosols and repellents to prevent nuisance, especially in urban and suburban areas, from culicid bites [10,20]. Most of these widely used mosquito control products contain at least pyrethroid insecticides [21]. According to Marcombe et al. [22], deltamethrin resistance in adult populations of *Ae. aegypti* was positively correlated to urbanization, deltamethrin outdoor applications and to the adult over-transcription of the genes *CYP9M9* and *GSTE7*. It is also possible that the high selection pressure exerted on the larval populations in periurban vegetable crops has increased resistance to insecticides [23]. The use of the same insecticides in urban vegetable crops and in public health (organophosphates, OP and pyrethroids) can contribute to polluting breeding sites, leading to faster development of resistance to these compounds [24]. The dispersion of household waste and empty packaging of pesticides in urban areas also allows resistance to be selected at the time of rainwater runoff. 

Also, the use of insecticides in cotton and vegetable growing areas was widely involved in the emergence of the crossed resistance DDT/pyrethroids as shown by Diabaté et al. [25] and Dabiré et al. [26] in *Anopheles* mosquitoes. Recently, a cross resistance DDT/pyrethroid was detected in *Aedes* populations of Ouagadougou and Bobo-Dioulasso (Unpublished data). Many studies across Africa have demonstrated the implication of genetic mechanisms in this cross-resistance DDT/pyrethroids within *Ae. aegypti* populations [27]. Several amino acid substitutions have been detected in *Ae. aegypti* voltage sensitive sodium channel gene (Vssc) [28]. Among them, only substitutions *V1016G, V1016I*, and *F1534C* have been shown to be strongly correlated with pyrethroid resistance [29,30,31,32]. Recently, Badolo et al. [33] showed that *F1534C* mutation was nearly fixed in semi-urban and urban areas of Burkina Faso but was far less common in rural areas, where the *V1016I* mutation frequency was also significantly lower. In addition, Badolo et al. [33] suggested the involvement of metabolic resistance mechanisms involving P450s, and perhaps esterases as a pre-exposure to PBO restored a substantial part of the susceptibility to permethrin and deltamethrin. Our study documents for the first time the variation of detoxifying enzymatic activities in the populations of *Aedes* of Ouagadougou and Bobo-Dioulasso. 

Interestingly, the wild populations of *Ae. aegypti* were found to be fully susceptible to chlorpyrifos-methyl during the three-year follow-up. In Burkina Faso, and likely elsewhere in continental Africa, the demonstration of sensitivity to organophosphates suggests that insecticides of this class are interesting options for controlling epidemics. GSTs and NSE activities might therefore complement the phenotypic effect of gene mutations towards increasing resistance levels to OP and broadening the resistance spectrum to unrelated compound. High levels of GST and NSE activities were observed in both Ouagadougou and Bobo-Dioulasso populations of *Ae. aegypti* [34]. According to Hemingway et al. [35], enhanced NSE activity may be involved in resistance to OP and carbamate (CM) in a number of arthropod species including mosquitoes, ticks, aphids and cockroaches. Hence, NSEs could further contribute to the phenotypic resistance to pyrethroid and carbamates in *Ae. aegypti* from Burkina Faso. In addition, there was evidence for an overall increase in cytochrome P450 activity in the samples from Ouagadougou, as compared to the Montpellier reference strain assessed simultaneously. That may explain the major role of cytochrome P450 in the metabolic resistance of *Aedes* populations from Ouagadougou. In this present study, we provided evidence that insecticide multi-resistance is a common phenotype within *Ae. aegypti* populations from Burkina Faso, while a previous study showed that target-site mutations are widespread [33]. 

## 5. Conclusions

As no efficient vaccine against dengue fever exists, vector control relies on insecticide use and remains the main strategy to reduce the spread of dengue fever outbreaks. In the current survey, *Ae. aegypti* populations were found to be fully resistant to pyrethroids and carbamates in the two main cities of Burkina Faso, West Africa. For the first time, detoxification enzymes were found to be involved in the insecticide resistance within *Ae. aegypti* populations in Burkina Faso. 

Despite this high resistance to pyrethroids and carbamates, organophosphate compounds remain effective against *Aedes* mosquitoes. These interesting data could be useful for health policy makers in their design strategies. Here, we pointed out the occurrence of metabolic resistance and how these results could impact the surveillance planning. 

## Data Availability

The datasets used during the current study are available from the corresponding author on request.

## Abbreviations

GST	Gluthatione S-transferase
*kdr*	Knockdown resistance
NS	Non-specific
NSE	Non-specific Esterase
PNPA	Para Nitro Phenyl Acetate
WHO	World Health Organization
